# Supplementary material for: Comparative multiomics analysis of cell physiological state after culture in a basket bioreactor
Source: Sci Rep. 2022 Nov 23;12:20161. doi: 10.1038/s41598-022-24687-4 (PMC9686226; doi:10.1038/s41598-022-24687-4)

23666075.1:14247607:14247670:-@NW_023666075.1:14246823:14247670:-@NW_023666075.1:14246823:1424

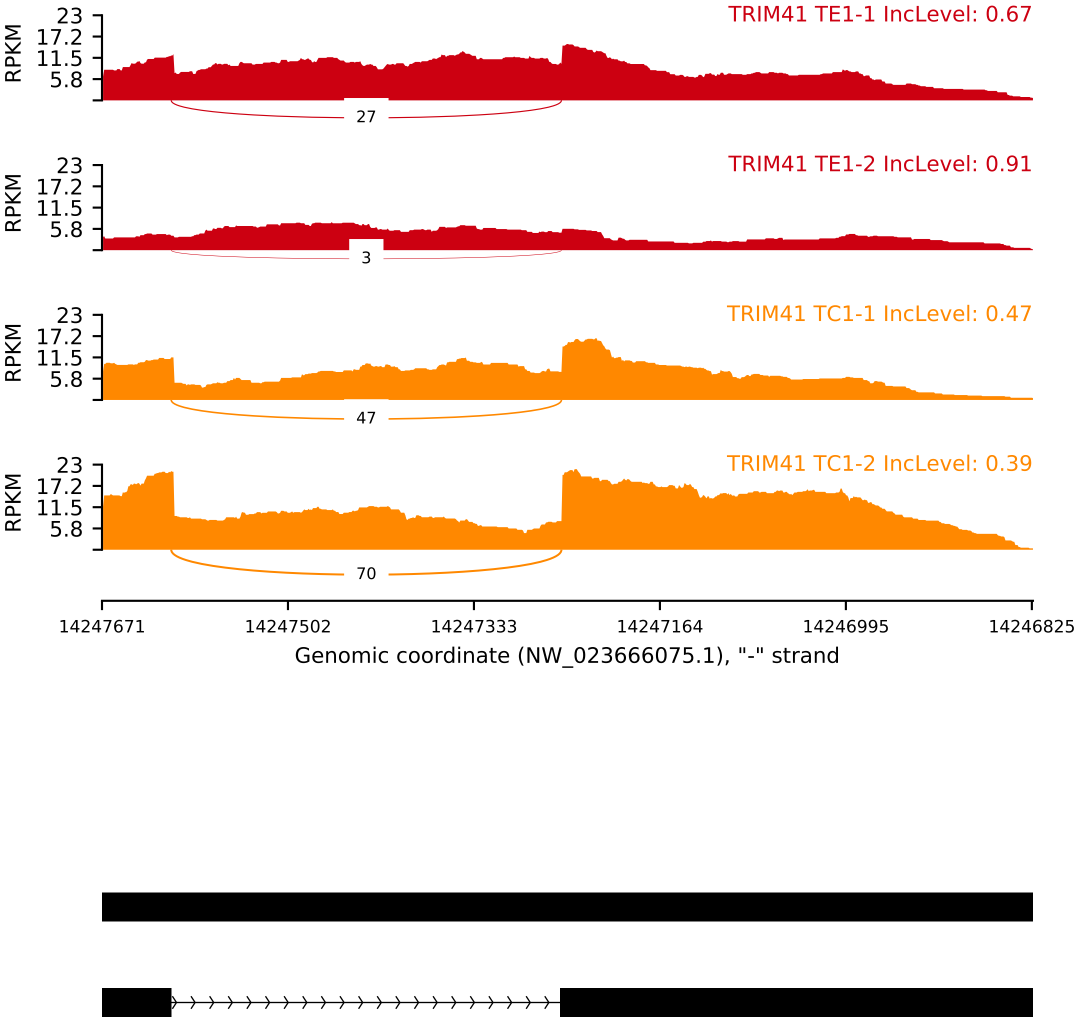

Supplement: Supplementary file 1 — Supplementary Information 1. [file 41598_2022_24687_MOESM1_ESM.zip › raw data/Transcriptomic raw data/7.AS/2.dasplot/TE1vsTC1/RI/18_TRIM41_NW_023666075.1_14247607_14247670_-@NW_023666075.1_14246823_14247670_-@NW_023666075.1_14246823_14247253_-.pdf]
